# Optimum N:P:K Ratio of Fertilization Enhances Tomato Yield and Quality Under Brackish Water Irrigation

**DOI:** 10.3390/plants14162496

**Published:** 2025-08-11

**Authors:** Lanqi Jing, Jianshe Li, Yongqiang Tian, Longguo Wu, Yanming Gao, Yune Cao

**Affiliations:** 1 College of Civil and Hydraulic Engineering, Ningxia University, Helanshan Xilu No. 489, Yinchuan 750021, China; jinglanqi0818@163.com (L.J.); 13709587801@163.com (J.L.); 2 College of Wine and Horticulture, Ningxia University, Helanshan Xilu No. 489, Yinchuan 750021, China; wlg@nxu.edu.cn; 3 College of Horticulture, China Agricultural University, Yuanmingyuan West Road No. 2, Haidian District, Beijing 100193, China; tianyq1984@cau.edu.cn

**Keywords:** brackish water, integrated nitrogen–phosphorus–potassium fertilization, tomato, quality, growth and development

## Abstract

Excessive or improper fertilization not only salinizes soil but also reduces crop yield and quality. The objective of this study was to determine the optimum N, P, and K levels capable of improving tomato fruit quality and reducing environmental pollution for tomato plants under brackish water irrigation conditions. The ‘Jingcai 8’ tomato was used as the research object, and an orthogonal experimental design was used to set up three nutritional factors of N, P, and K. Each factor was set at three levels: N (mmol·L^−1^): 2.00 (N1), 4.00 (N2), and 8.00 (N3); P (mmol·L^−1^): 0.67 (P1), 1.33 (P2), and 2.00 (P3); K (mmol·L^−1^): 8.00 (K1), 12.00 (K2), and 16.00 (K3). The effects of different levels of N, P, and K on plant growth indexes, root vigor and antistress enzymes, biomass and nutrients of plants and fruits, yield, quality, soil nutrients, and soil enzymes were investigated, and metabolomic measurements were performed on treatments ranked first (N:P:K ratio was 2:1.33:12) and ninth (N:P:K ratio was 8:1.33:8) for overall quality. In general, a N concentration of 8 mmol·L^−1^ promoted plant vegetative growth and plant biomass accumulation by promoting the accumulation of aboveground nitrogen content, but it reduced the weight of single fruit and tomato quality due to an increase in soil EC and pH. In contrast, 0.67 mmol·L^−1^ of P and 12 mmol·L^−1^ of K were able to promote both plant vegetative growth and tomato quality formation. In addition, 0.67 mmol·L^−1^ of P enhanced soil nutrient availability and enzyme activity, while 16 mmol·L^−1^ of K reduced nutrient availability and enzyme activity and increased soil EC. The concentrations of ferulic acid, cinnamic acid, caffeic acid, coumarin, and (-)-epigallocatechin were generally higher in tomatoes from the T2 treatment (N:P:K ratio was 2:1.33:12) than in those from other treatments. Together, the optimum N:P:K ratio (2:1.33:12) of fertilization enhances tomato yield and quality under brackish water irrigation.

## 1. Introduction

With an increasing shortage of freshwater resources in the past decade, the utilization of brackish water has received more and more attention. The rational utilization of brackish water has become one of the most important ways to alleviate water shortages. While alleviating freshwater shortage, brackish water irrigation has a significant impact on tomato quality. Irrigation with brackish water (electrical conductivity EC ≤ 3 dS/m) can significantly increase the contents of total soluble solids, sugar–acid ratio, organic acids, and vitamin C in tomato fruits, improving flavor and nutritional quality [1]. Moderate salt stress (e.g., 2–4 g/L) under spring cultivation conditions helps promote tomato growth and quality improvement, but continuous high-salt irrigation or autumn cultivation may inhibit crop growth and yield [2]. Molecular and transcriptomic studies further revealed that brackish water irrigation promotes the accumulation of soluble sugars such as glucose and fructose by regulating the expression of genes related to sugar metabolism [3]. In addition, reasonable measures such as combining organic materials (e.g., filter cake) or intercropping can alleviate soil salt accumulation and improve soil quality and tomato yield, while maintaining high fruit quality [4]. Therefore, the scientific use of brackish water, especially within an appropriate salinity range and under reasonable irrigation management, is an effective way to improve tomato fruit quality and achieve high-quality production.

In the arid regions of northwest China, brackish water is often used as an important irrigation water source in agricultural irrigation, and its main ionic components include Na^+^, Ca^2+^, Mg^2+^, Cl^−^, SO_4_^2−^ and HCO_3_^−^ [5,6]. The four salts selected in this experiment (NaHCO_3_, MgSO_4_·7H_2_O, CaCl_2_, and NaCl) can accurately reproduce the common main ion types and their proportions in brackish water in arid and semi-arid regions and effectively simulate the chemical characteristics of irrigation water in these regions. The concentrations of ions such as Na^+^ and Cl^−^, as well as the electrical conductivity (EC 2.0 mS·cm^−1^), are highly consistent with the typical ion range and electrical conductivity interval (1.0–3.0 mS·cm^−1^) of actual brackish water used for agricultural irrigation in the arid regions of Northwest China [7,8]. Relevant studies have shown that when the EC value is 2.0 mS·cm^−1^, the concentrations of major ions such as Na^+^ and Cl^−^ are consistent with those in actual irrigation water bodies. Moreover, this level of electrical conductivity is considered suitable for farmland irrigation as it can ensure crop growth while reflecting the real salt stress caused by brackish water irrigation [7,8]. Therefore, the ion composition and electrical conductivity of the brackish water used in this experiment can effectively simulate the typical conditions of brackish water irrigation in local agricultural production, providing a reliable basis for related research.

Tomato (*Solanum lycopersicum* L.) occupies an important position in the world and is rich in many essential vitamins and minerals. With improved living standards and health awareness, people’s demand for tomatoes gradually changes from quantity to quality, and they begin to pay more attention to the intrinsic quality of tomatoes. Nitrogen (N), phosphorus (P), and potassium (K) are important nutrients for improving crop yield and quality during crop growth and development [9,10]. N plays an important role in regulating fruit quality. A reasonable application of N fertilizer can significantly promote photosynthesis in crop plants and increase fruit yield, polyphenols, and other substances [11]. Cheng et al. [12] analyzed the effect of N supply on the yield and fruit quality of tomato and showed that the optimum application of N resulted in relatively high tomato yields, vitamin C, sugar–acid ratio, soluble sugar, and soluble solid as compared to no N supply. P is an essential nutrient for plant growth and development and plays a vital role in cellular metabolism. Phosphorus is an essential nutrient for plant growth and development. Liu et al. [13] showed that under a moderate phosphorus concentration (such as 70% irrigation amount), tomatoes have the highest sugar–acid ratio, soluble solids, and flavor substance content, and the best water and fertilizer use efficiency. K is the second-most abundant element in plant tissues, and K improves fruit water absorption and quality [14]. K plays an important role in plant development by promoting cell elongation, plant water management, and carbohydrate synthesis [15]. Almeselmani et al. [16] studied the effect of different K levels on the quality of tomato and showed that fruit yield increased with an increase in K concentration, while protein, ascorbic acid, lycopene, total soluble solids, reducing sugar, and titratable acidity were significantly increased in the fruit when K concentration was increased up to 300 mg·L^−1^. In addition, in crops such as potato, wheat, and rice the combined application of NPK can significantly increase root biomass, leaf photosynthetic capacity, antioxidant enzyme activity, and nitrogen use efficiency, thereby resulting in higher yields and better quality [17,18]. There are synergistic effects between N, P, and K. The interactions between nitrogen and potassium, as well as between nitrogen and phosphorus, are particularly important for crop growth, while the interaction between phosphorus and potassium may exhibit antagonism [19]. Long-term field experiments have also found that the efficient utilization of potassium fertilizer depends on the sufficient supply of nitrogen and phosphorus, and the balanced application of the three helps maintain soil nutrient balance and sustainable high crop yields [18].

Previous studies have clearly demonstrated the critical role of nitrogen, phosphorus, and potassium (NPK) in crop growth, and the form of nutrient supply directly affects their utilization efficiency. From the early application of single elements to the later combined application of compound fertilizers, fertilization technology has been continuously developing towards precision. Among these, NPK nutrient solutions, by dissolving and blending nutrients such as nitrogen, phosphorus, and potassium, form a liquid nutrient system that is more easily absorbed by crops. With the characteristic of flexibly adjusting nutrient supply during critical stages of crop growth, they show broad application prospects in various cultivation practices [20]. As a core fertilization method in modern facility agriculture and various cultivation patterns, NPK nutrient solutions are not only one of the most effective ways to apply NPK efficiently but also can dynamically adjust nutrient ratios according to crop needs and growth stages to achieve precise nutrient management [20]. They provide a new solution for realizing precise nutrient supply and dynamic regulation and have been widely proven to significantly improve the yield and quality of crops such as tomatoes. In recent years, research has focused on the precise ratio of NPK nutrient solutions, application methods (such as drip irrigation, foliar spraying, etc.), synergistic application with organic fertilizers, biostimulants, trace elements, nano-fertilizers, microbial fertilizers, etc., as well as their impacts on quality indicators of tomato fruits such as nutrients (e.g., soluble sugars, vitamin C, lycopene, β-carotene), flavor, and antioxidants [21,22,23,24,25]. Some studies have confirmed that NPK nutrient solutions can not only enhance key nutritional components of tomatoes but also improve fruit flavor, firmness, antioxidant capacity, and freshness retention [21,23]. Moreover, the integrated management mode of NPK nutrient solutions with organic fertilizers, biological fertilizers, etc., can further improve nutrient use efficiency, enhance soil health, strengthen crop stress resistance, and achieve high yield, high quality, and sustainable production [24]. Due to the differences in NPK requirements among different crops and growth environments and the fact that the effects of different NPK ratios and application frequencies on tomato quality are dependent on varieties and environments [22], scientifically determining and dynamically adjusting the NPK ratio in nutrient solutions and reasonably optimizing the formula and management mode are crucial for improving crop quality and achieving efficient and sustainable production.

In recent years, metabolomics has become a core technology for analyzing the regulation of tomato fruit quality by NPK (nitrogen, phosphorus, and potassium) nutrient solutions. Through high-throughput metabolite detection and multi-omics integration, researchers have revealed the regulatory mechanisms of NPK nutrient solutions and their management methods on key quality-related metabolites in tomatoes, such as sugars, organic acids, amino acids, flavonoids, carotenoids, and volatile compounds [26,27]. Organic NPK nutrient solutions can not only improve the flavor and nutrition of tomatoes but also promote the accumulation of aromatic substances and antioxidants [26]. Different NPK ratios, application methods (such as rhizosphere application, foliar spraying, slow-release fertilizers, etc.) and their combined application with biostimulants, microbial fertilizers, nano-fertilizers, etc., can significantly improve tomato fruit quality indicators such as soluble sugars, vitamin C, lycopene, and β-carotene by regulating metabolic pathways [21]. In addition, the impact of NPK nutrient solutions on tomato quality is also regulated by factors such as varieties and environments (e.g., temperature, light, salt stress) [27]. In summary, metabolomics provides a theoretical basis and technical support for optimizing NPK nutrient solution formulations, improving tomato quality, and achieving precise cultivation management.

Although many studies have revealed the effects of N, P, and K fertilizers on plant growth, crop yield, and quality, the effects of nutrient solutions with N, P, and K under brackish water irrigation on crop yield and quality have been slightly understudied. The objective of this study was to determine the optimal levels of N, P, and K that match the needs of tomato plants under brackish water irrigation conditions. We measured soil nutrients, tomato physiological indices, and tomato metabolites treated by nutrient solutions with different N, P, and K ratios under brackish water irrigation conditions.

## 2. Materials and Methods

### 2.1. Time and Place of Testing

The experiment was conducted from 22 August 2022 to 10 January 2023 in a solar greenhouse located at the Research and Development Center of Agricultural Science and Technology Park, Helan County, Yinchuan City, Ningxia (106.19° E, 38.35° N). The altitude of the test site is 1110.14 m, the average annual temperature is 8.5 °C, the frost-free period is about 155 days, the average annual sunshine hours are about 2800–3000 h, the average annual precipitation is about 200 mm, and the regional climate is typical temperate continental climate.

### 2.2. Experimental Design

The test tomato was the pink-fruited, early-maturing variety “Jingcai No. 8”, which was provided by Beijing Modern Farmer Seedling Science and Technology Co. The experiment was conducted using soil cultivation. There were 5 spikes of fruit for each tomato plant. Plants were irrigated with freshwater on days 1–10 after transplanting.

The brackish water (EC = 2.0 mS·cm^−1^) used in the experiment was prepared by adding four industrial salts (NaHCO_3_ 0.314, MgSO_4_·7H_2_O 0.142, CaCl_2_ 0.168, and NaCl 0.302 g·L^−1^) to the original greenhouse fresh groundwater (EC = 0.69 mS·cm^−1^). Brackish water has a pH of 7.2. An orthogonal experimental design was used to set three nutrient factors including N, P, and K. Each factor was set at three levels. N was set as low N (N1), medium N (N2), and high N (N3) at 2, 4, and 8 mmol·L^−1^, respectively; P was set as low P (P1), medium P (P2), and high P (P3) at 0.67, 1.33, and 2 mmol·L^−1^, respectively; K was set as low K (K1), medium K (K2), and high K (K3) at 8, 12, and 16 mmol·L^−1^. The experiment was arranged in randomized blocks with nine treatments (Table 1) and three replications per treatment. The plants were spaced 46 cm apart and the rows were spaced 75 cm apart. The plot area was 6 m^2^. The bulk elements used in the experiment were based on Ca(NO_3_)_2_-4H_2_O 1181.25, KNO_3_ 404.50, NH_4_H_2_PO_4_ 76.5, and MgSO_4_-7H_2_O 493 mg·L^−1^ and were configured according to the N, P, and K ratios of the nine treatments with NH_4_H_2_PO_4_, KH_2_PO_4_, KNO_3_, K_2_SO_4_, Ca(NO_3_)_2_-4H_2_O, CaCl_2_, and MgSO_4_-7H_2_O salts. The trace elements used in the experiment were the following generalized formulations: Fe 3.0, B 0.5, Mn 0.5, Zn 0.05, Cu 0.02, and Mo 0.01 mg·L^−1^. Each treatment used a black bucket with a volume of about 170 L to store brackish water and nutrient solution, and a 200 w water pump was placed in the bucket and supplied by drip irrigation under the membrane. A total of 20.4 mL of micronutrients were added to each 170 L of nutrient solution, 2 drip irrigation tapes were installed in each plot, and the irrigation time is shown in Table 2. The management was the same except for the different nutrient solution for watering. The physical and chemical properties of the soil are shown in Table 3.

### 2.3. Determination of Indicators

A steel tape measure was used to determine plant height, vernier calipers were used to determine stem thickness, and a steel tape measure was used to determine the length and width of the leaves and to calculate the leaf area [28]. The root vigor of representative plants of tomato at late growth stage was determined using the triphenyl tetrazolium chloride (TTC) method [29]. Malondialdehyde (MDA) was determined by the thiobarbituric acid method; superoxide dismutase (SOD) by the NBT reduction method; catalase (CAT) by the ultraviolet absorption method; peroxidase (POD) by the colorimetric method; and proline (Pro) by the acid ninhydrin method [29]. The fresh mass of each part of the plant and fruit was weighed, and the dry mass of each part and fruit was weighed after drying to a constant weight. Total nitrogen was determined by the Kjeldahl method; total phosphorus was determined by the molybdenum antimony resistance colorimetric method; and total potassium was determined by the flame photometric method [30]. The weight and number of fruits harvested in each plot were recorded, and the average fruit weight and average yield per plant were calculated and finally converted into yield per hectare. During the tomato bloom, six fresh fruits from each treatment were randomly selected for quality determination. Soluble sugars were determined by colorimetric analysis with anthrone reagent [31]; titratable acids were determined by acid–base titration reactions [31]; total vitamin C was determined by the molybdenum blue colorimetric method [31]; and soluble solids were determined with a handheld digital display saccharimeter (TD-45) [31]. Soil total nitrogen and available nitrogen were determined using the Kjeldahl method; soil total phosphorus was decocted with HClO_4_-H_2_SO_4_ and determined by the molybdenum antimony colorimetric method; soil available phosphorus was determined by the molybdenum antimony colorimetric method; and soil available potassium was determined by the flame photometric method [30]. Soil urease, alkaline phosphatase, sucrase, and catalase were determined by the sodium phenol–sodium hypochlorite colorimetric method, sodium benzene diphenyl phosphate colorimetric method, 3,5-dinitrosalicylic acid colorimetric method, and potassium permanganate titrimetric method, respectively [30]. Soil pH and EC values were determined with a pH meter and EC meter (soil: distilled water = 1:10) [30].

### 2.4. Metabolite Testing and Data Analysis

PCA (KMO > 0.5, *p* < 0.01) was performed on the results of tomato quality measurements. The highest and lowest ranked treatments were selected for sampling and frozen in liquid nitrogen and stored at −80 °C for metabolomics analysis. Six replicates were set for each sample. The samples were entrusted to Shanghai Majorbio Bio-pharm Technology Co., Ltd. (Shanghai, China) for metabolite determination and data analysis. The raw data were pre-processed with Majorbio’s own software Version 1.0.0, Version 1.6.2, and Release 1 May 2017. Univariate statistical analysis (*t*-test) combined with orthogonal partial least squares–discriminant analysis (OPLS-DA) and fold-change values (FCs) were used to screen for differential metabolites, with a screening condition of *p* < 0.05 and VIP > 1. FC > 1 indicated an increase in metabolites, and FC < 1 indicated a decrease in metabolites.

### 2.5. Data Analysis of Physiological Indicators

Data statistics, analysis, and graphing were performed using SPSS 23.0 software and Excel 2018, and analysis of variance (ANOVA) was performed using Duncan’s method for the significance of difference test (*p* < 0.05).

## 3. Results

### 3.1. Growth Indices and Root Vigor

**Figure 1:**
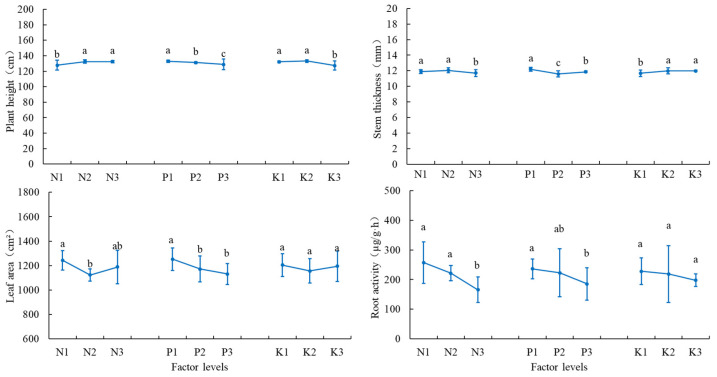
Growth indices and root vigor at different N, P, and K levels. The error bars represent the standard deviation (SD) of the mean values. Different letters over each bar under each factor and each level represent a significant difference at *p* ≤ 0.05.

The effects of different treatments on tomato growth indexes varied significantly (Table 4). Tomato plant height and stem thickness were at their maximum under the T4 treatment (63 cm and 12.44 cm, respectively) and were significantly higher than those under the T2 and T5 treatments. Tomato leaf area was at its maximum under the T7 treatment (1310.58 cm^2^) and was significantly higher than that under the T4, T5, and T6 treatments. The plant height and stem thickness generally showed the lowest values, respectively, at N1, P3, and K3 and N3, P2, and K1 levels, while leaf area showed the lowest value at N2 and P3 levels (Figure 1).

Root vigor is an important indicator of plant root health and growth and one of the most important factors in plant productivity and nutrient uptake. Tomato root vigor was at its maximum under the T2 treatment (304.09 μg·g^−1^·h^−1^), and it showed the lowest value at N3 and P3 levels (Figure 1).

### 3.2. Leaf Biochemical Indicators of Salt Tolerance

**Figure 2:**
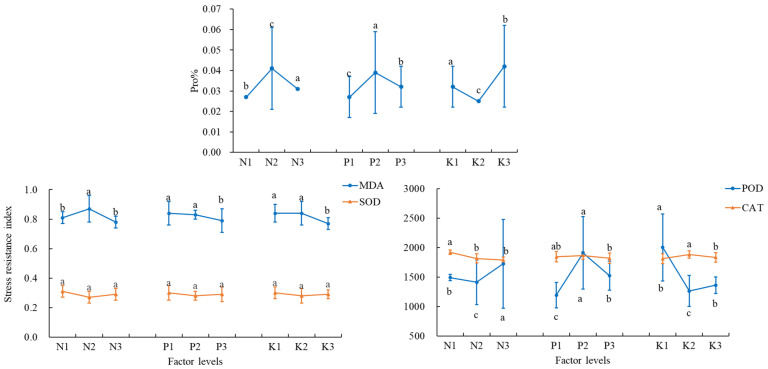
Indicators of leaf stress tolerance at different N, P, and K levels. The error bars represent the standard deviation (SD) of the mean values. Different letters over each bar under each factor and each level represent a significant difference at *p* ≤ 0.05.

MDA was highest in the T4 treatment (0.94 µmol/g FW); SOD was highest in the T1 treatment (0.34 U·mg^−1^·min^−1^); POD was highest in the T8 treatment (2727.78 U·(gF·min^−1^)^−1^) and was significantly (*p* < 0.05) higher than all other treatments; and Pro content was highest in the T5 treatment (0.061%), and was significantly (*p* < 0.05) higher than all other treatments. CAT activity was highest in the T2 treatment (1938.25 U·g^−1^·min^−1^; Table 5). In addition, MDA showed the highest values at N2 and P2 levels, while Pro showed the highest values at N2, P2, and K3 levels. As for POD and CAT, the optimal levels were N3, P2, and K1 and N1, P2, and K2, respectively (Figure 2).

### 3.3. Fruit and Plant Biomass, Nutrient Concentration, and Yield in Tomato

#### 3.3.1. Biomass Production and Yield of Tomato Fruits and Plants

**Figure 3:**
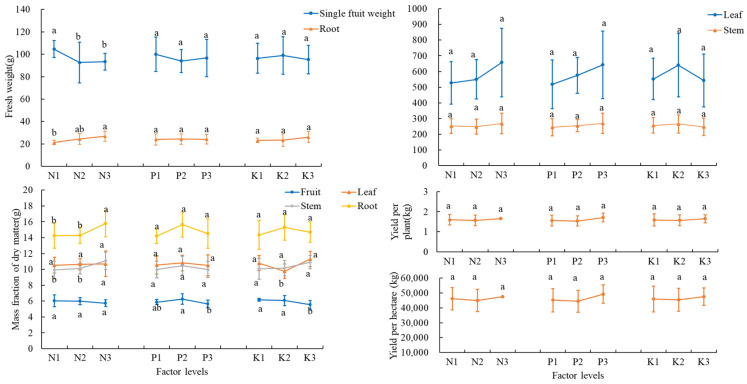
Yield and biomass at different N, P, and K levels. The error bars represent the standard deviation (SD) of the mean values. Different letters over each bar under each factor and each level represent a significant difference at *p* ≤ 0.05.

The single fruit weight was at its maximum in the T7 treatment (106.21 g) and was significantly higher than that in the T5 treatment (*p* < 0.05). There were no significant differences among the treatments for tomato yield (Table 6). However, the N1 level exhibited a higher single fruit weight as compared to the N2 and N3 levels (Figure 3).

Both leaf and stem fresh weights were heaviest in the T9 treatment, reaching 858.15 g and 441.76 g, respectively, with significant (*p* < 0.05) differences between them and those in the T3, T4, and T8 treatments. The root fresh weight was highest in the T5 treatment at 28.6 g, which was significantly higher than that in the T2 treatment (*p* < 0.05) (Table 6). For the root fresh weight, the N3 level had the best effect (Figure 3).

The dry matter mass fraction of tomato was highest in the T2 treatment at 6.82%, which was significantly different from all treatments except T6 (*p* < 0.05); the dry matter mass fraction of leaves was highest in the T3 treatment at 11.68%, which was significantly different from the T1, T2, and T9 treatments (*p* < 0.05); and the dry matter mass fractions of both stems and roots of the plants were highest in the T9 treatment at 11.15% and 16.17% (Table 7). For tomato dry weight, P2 and K1 levels had the best effect; for leaf dry weight, the K3 level had the best effect; and for stem and root dry weight, the N3 level had the best effect (Figure 3).

#### 3.3.2. Plant and Fruit Nutrient Concentrations

**Figure 4:**
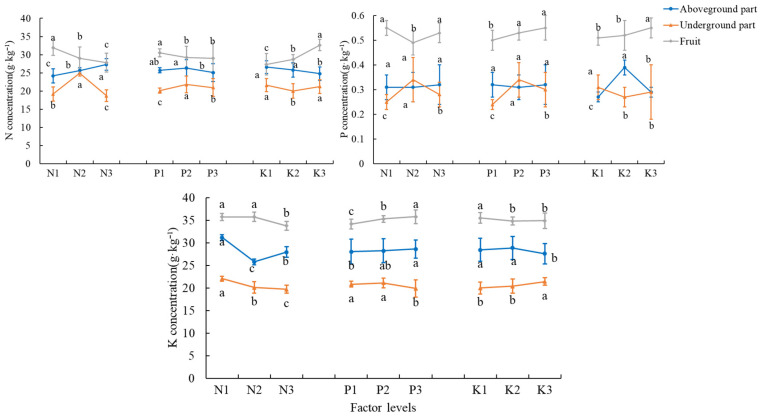
Plant and fruit nutrient concentration at different N, P, and K levels. The error bars represent the standard deviation (SD) of the mean values. Different letters over each bar under each factor and each level represent a significant difference at *p* ≤ 0.05.

The total nitrogen concentration in the underground part of the plant was highest in the T5 treatment at 27.35 g·kg^−1^; the total nitrogen concentration in the aboveground part of the plant was highest in the T8 treatment at 28.56 g·kg^−1^; and the total nitrogen concentration of tomato fruits was highest in the T3 treatment at 34.48 g·kg^−1^ (Table 7). The levels of N2, P2, and K3 had the best effect for total nitrogen in the underground part of the plant; the levels of N3, P2, and K1 had the best effect for total nitrogen in the aboveground part of the plant; and the levels of N1, P1, and K3 had the best effect for total nitrogen in tomato fruits (Figure 4).

The concentration of total phosphorus underground was highest in the T5 treatment, reaching 0.43 g·kg^−1^; the concentration of total phosphorus aboveground was highest in the T9 treatment, reaching 0.43 g·kg^−1^; and the concentration of total phosphorus in the fruit was highest in the T3 treatment, reaching 0.59 g·kg^−1^ (Table 7). For underground total phosphorus, the levels of N2, P2, and K1 had the best effect; for aboveground total phosphorus, level K2 had the best effect; and for tomato fruit total phosphorus, the levels of N1, P3, and K3 had the best effect (Figure 4).

The total potassium concentration underground was highest in the T3 treatment, amounting to 22.4 g·kg^−1^; the total potassium concentration in the aboveground part of the plant was highest in the T1 treatment, amounting to 31.69 g·kg^−1^; and the total potassium concentration of tomato fruits was highest in the T6 treatment, amounting to 36.57 g·kg^−1^ (Table 7). The levels of N1, P2, and K3 had the best effect for total potassium in the underground part of the plant; the levels of N1, P3, and K2 had the best effect for total potassium in the aboveground part of the plant; and the levels of N1, P3, and K1 had the best effect for total phosphorus in tomato fruits (Figure 4).

### 3.4. Fruit Quality and DEMs Analysis

**Figure 5:**
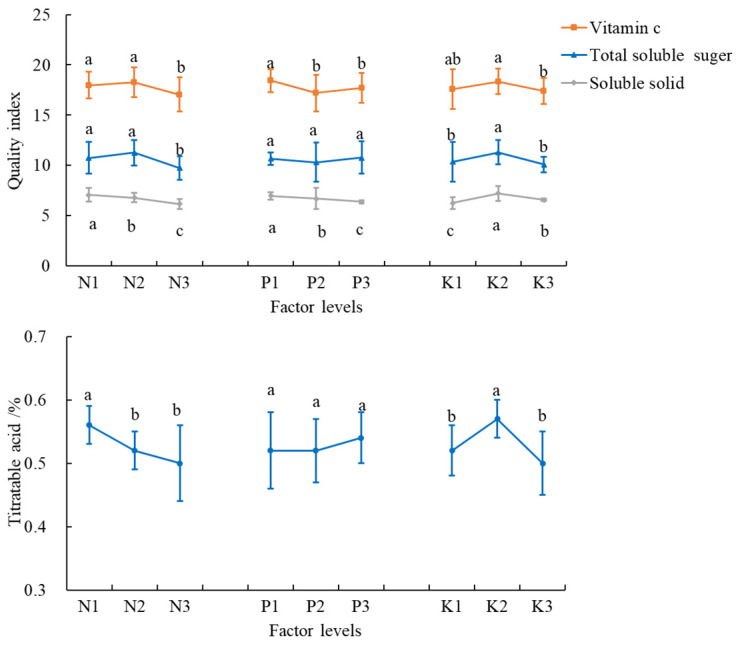
Tomato quality at different N, P, and K levels. The error bars represent the standard deviation (SD) of the mean values. Different letters over each bar under each factor and each level represent a significant difference at *p* ≤ 0.05.

All quality indicators were lowest in the T8 treatment. Both VC and soluble sugar concentrations were highest in the T6 treatment with 19.41 mg·100g^−1^·FW, 12.74%. Soluble solids were highest in the T2 treatment at 7.97%, and all the differences with other treatments were significant. Titratable acid was highest in the T9 treatment at 0.58% (Table 8). For VC and soluble sugars, the levels of P1 and K2 and N2 and K2 had the best effects; for soluble solids and titratable acids, the levels of N1, P1, and K2 and N1 and K2 had the best effects (Figure 5).

PAC (KMO > 0.5, *p* < 0.005) was performed for the four qualities of soluble solids, VC, total soluble sugars, and titratable acids. A total of two principal components were extracted; the eigenvalues of principal component one and principal component two were 2.629 and 1.058, respectively; the contribution rates were 65.73% and 26.453%, respectively; the weights were 0.713 and 0.287, respectively; and the cumulative contribution rates were 65.73% and 92.183%, respectively, from which it can be derived that the equation of principal component one was Y1 = 0.581 × 1 + 0.577 × 2 + 0.543 × 3 + 0.185 × 4, the equation of principal component two was Y2 = −0.270 × 1 − 0.227 × 2 + 0.221 × 3 + 0.909 × 4, X1 represented VC, X2 represented soluble sugar, X3 represented soluble solids, X4 represented titratable acid, and finally, the total equation used to calculate the value of the integrated principal component of quality was Y = 0.65730Y1 + 0.26453Y2. The ranking showed that T2 was the highest and T8 was the lowest (Table 8).

**Figure 6:**
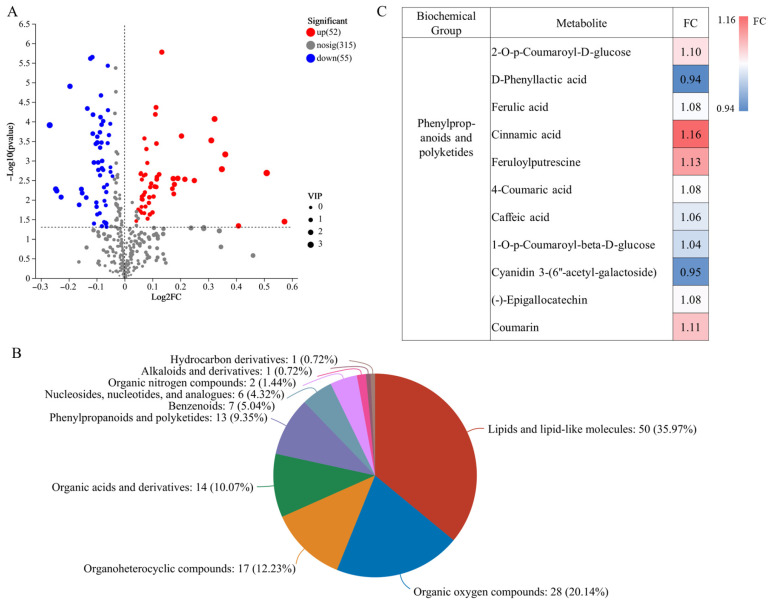
Volcano plots of DEMs. The horizontal coordinate is the value of the fold change in the difference in metabolite expression between the two groups, i.e., log2FC, and the vertical coordinate is the value of the statistical test of the difference in metabolite expression change, i.e., −log10 (*p*-value) value, where the higher the value, the more significant the difference in expression. Each point in the graph represents a specific metabolite, and the size of the point indicates the Vip value. The more to the left, right, and upper sides of the point, the more significant the expression difference (**A**) classification of DEMs compounds, (**B**) changes in the concentration of phenylpropanoids and polyketide, FC > 1 indicated an increase in metabolites and FC < 1 indicated a decrease in metabolites (**C**).

Metabolites were screened according to differential metabolite screening criteria. T2 vs. T8 screened 167 (77 increased metabolites and 90 decreased metabolites) significant DEMs (Figure 6A). The DEMs were divided into nine main groups, including lipids and lipid-like molecules, organic acids and derivatives, organic oxygen compounds, organic hete-ocyclic compounds, benzenoids, and phenylpropanoids and polyketide, among others (Figure 6B). Among the phenylpropanoids and polyketide compounds, Ferulic acid, Cinnamic acid, Caffeic acid, Coumarin, and (-)-Epigallocatechin, which are closely related to quality, were significantly increased (Figure 6C). Therefore, it is possible that the increase in the concentration of these substances led to the higher overall quality of the T2 treatment than that of T8.

### 3.5. Soil Nutrients, pH, EC, and Soil Enzymes

#### 3.5.1. Soil Nutrients, pH, and EC

**Figure 7:**
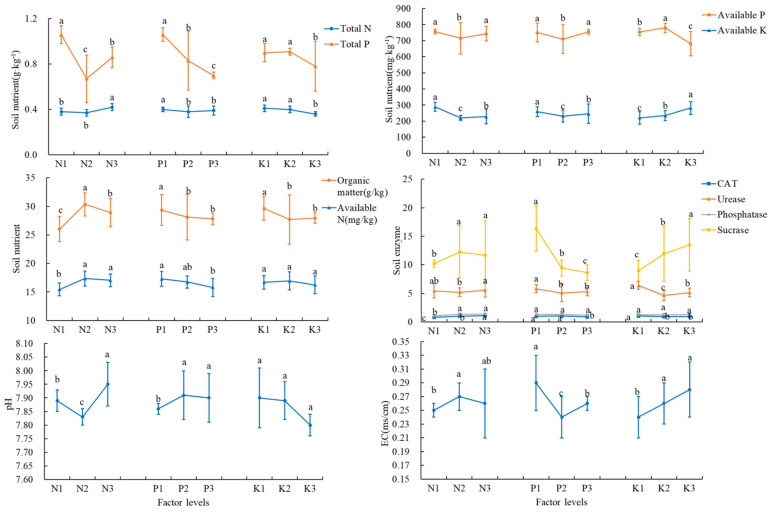
Soil nutrients, enzyme activities, pH, and EC at different N, P, and K levels. The error bars represent the standard deviation (SD) of the mean values. Different letters over each bar under each factor and each level represent a significant difference at *p* ≤ 0.05.

Soil total N concentration was highest in the T8 treatment at 0.45 g·kg^−1^; the total P concentration was highest in the T8 treatment at 0.97 g·kg^−1^ and reached a significant difference (*p* < 0.05) with all other treatments; available N was highest in the T4 treatment at 18.6 mg·kg^−1^; available P was highest in the T4 treatment at 815.9 mg·kg^−1^, which was significantly different from all other treatments (*p* < 0.05); and available K was highest in the T3 treatment at 324.6 mg·kg^−1^, which was significantly different from all other treatments (*p* < 0.05). Soil organic matter concentration was highest in the T4 treatment at 32.99 g·kg^−1^; EC was highest in the T4 treatment at 0.32 ms·cm^−1^; and pH was highest in the T8 treatment at 8.02 (Table 9). For total N, available N, and available P concentrations, N3, P1, and K2 levels were the best; for total P and available K, N1 and P1 levels were the best, and level K3 had the best effect on available K but had a poor effect on total P; for organic matter and pH, N2, P1, and K1 levels were the best; and for EC, N1, P2, and K1 levels were optimal (Figure 7).

#### 3.5.2. Soil Enzyme Activities

Both soil catalase and soil urease activities were highest in the T8 treatment, reaching 1.45 mg·g^−1^ and 7.05 mg·g^−1^, respectively, and reaching significant differences (*p* < 0.05) with other treatments. Soil alkaline phosphatase concentration was highest in the T4 treatment at 1.50 mg·g^−1^, which differed significantly (*p* < 0.05) from the T1, T2, T3, and T7 treatments. Sucrase activity was highest in the T7 treatment at 19.62 mg·g^−1^ and reached a significant difference (*p* < 0.05) with all other treatments (Table 10). For catalase, alkaline phosphatase, and sucrase, the levels of N3, P1, and K2 were optimal; for urease, the levels of N1, P1, and K2 were optimal (Figure 7).

#### 3.5.3. Correlation Analysis of Soil Nutrients and Soil Enzymes

**Figure 8:**
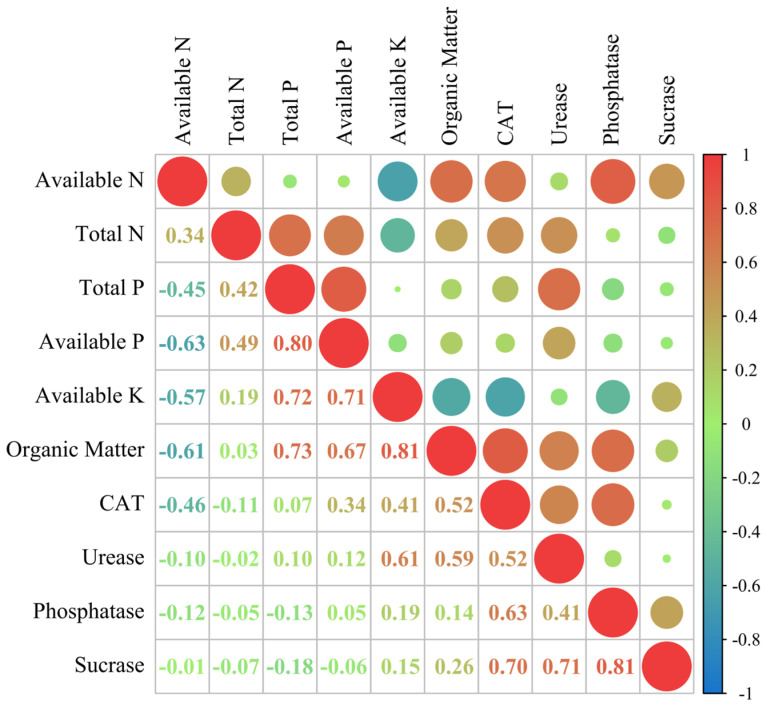
Correlation analysis of soil nutrients and soil enzymes.

From Pearson correlation analysis (Figure 8), soil available P showed a highly significant positive correlation (*p* < 0.01) with total *p*, with a correlation coefficient of 0.809; avail-able N showed a significant positive correlation (*p* < 0.05) with organic matter and catalase, with correlation coefficients of 0.712 and 0.669, respectively; and organic matter showed a highly significant positive correlation (*p* < 0.01) with catalase, with a correlation coefficient of 0.810. Furthermore, total P was significantly and positively correlated (*p* < 0.05) with urease, with a correlation coefficient of 0.707 and alkaline phosphatase was significantly and positively correlated (*p* < 0.05) with available N, organic matter, and catalase, with correlation coefficients of 0.796, 0.712, and 0.720, respectively.

### 3.6. Comprehensive Evaluation of PCA

PCA was used to comprehensively evaluate the effects of nutrient solution N, P, and K on growth and development, yield, and quality of tomato under brackish water irrigation. Root vigor, indicators of resilience other than MDA, fruit and plant dry weight and nutrients, tomato yield per hectare, tomato quality, soil nutrients, and soil enzymes were selected for principal component analysis. A total of eight principal components were extracted, and the eigenvalues and contribution rates of each principal component are shown in Table 11. Furthermore, the cumulative contribution rate of the eight extracted principal components is 100%. The F-value was the composite principal component value and the tomato with the T1 treatment had the highest principal component value of 1.35, while the tomato with the T5 treatment had the lowest F-value of −1.72, which was the worst performance in the composite evaluation (Table 12).

## 4. Discussion

### 4.1. Growth Indices and Root Vigor

Leaf area is influenced by many factors, and nitrogen is one of them. Lawlor et al. [32] showed that N pigments can affect leaf growth by increasing photosynthetic area through the synthesis of proteins involved in cell growth, cell division, and synthesis of the cell wall and cytoskeleton. In addition, leaf area is also affected by leaf nitrogen concentration, and Yang et al. [33] showed that the leaf area of crops increased with increasing nitrogen content. In this study, N3 level was able to promote tomato leaf area growth, similar to the results of Vos [34]. Meanwhile, aboveground total nitrogen concentration increased gradually with an increase in N concentration, similar to the results of the above study. In addition, the results of this experiment also showed that the nutrient growth of tomato plants was maximized at both a P concentration of 0.67 mmol·L^−1^ and a K concentration of 12 mmol·L^−1^, similar to the findings of Shu [35], Whitcher [36], and Kim [37].

Plant root vigor is strongly influenced by high or low N fertilizer application. In this study, root vigor was inhibited when N was increased to 8 mmol·L^−1^, similar to the findings of Chen [38] and Li [39]. In addition, 0.67 mmol·L^−1^ of P promoted root vigor in this study. Liao et al. [40] showed that root vigor could be significantly increased at appropriate phosphorus dosages (i.e., 0.9 and 1.2 mmol·L^−1^), and root vigor was significantly decreased when the phosphorus dosage was lower than 0.6 mmol·L^−1^ or higher than 1.5 mmol·L^−1^, which was similar to the results of the present study.

### 4.2. Leaf Biochemical Indicators of Salt Tolerance

Many studies have shown that a moderate increase in N, P, and K in nutrient solution can increase the concentration of Pro, SOD, POD, etc., in crop leaves [41,42,43]. In this study, Pro concentration was enhanced when the N concentration was increased from 2 to 4 mmol·L^−1^, and decreased when the N concentration exceeded 4 mmol·L^−1^; the activities of POD, Pro, and CAT enzymes were enhanced and the MDA concentration decreased when the P concentration was increased from 0.67 to 1.33 mmol·L^−1^, and the activities of each enzyme were inhibited when the concentration of P exceeded 1.33 mmol·L^−1^, similar to the results of Wang’s [44] and Liao’s [40] studies mentioned above. In this study, 8 mmol·L^−1^ of K was not favorable for proline concentration and catalase activity, but it increased MDA and POD concentration; inconsistencies with the results of Chen et al. [45] may be due to different cultivation media and potassium setting concentrations.

### 4.3. Fruit and Plant Biomass, Nutrient Concentration, and Yield in Tomato

Astuti et al. [46] in their study on crop biomass with different NPK applications showed that the application of NPK fertilizer significantly increased the dry matter mass of the crop as compared to that of the crop with no NPK fertilizer. Biemond et al. [47] showed that N fertilizers of 2.5, 8.0, and 16.0 gN·plant^−1^ had a significant effect on the aboveground biomass of the crop, with the higher doses of 8.0 and 16.0 gN·plant^−1^ resulting in a four-fold increase in aboveground biomass compared to 2.5 g N·plant^−1^. In this study, 8 mmol·L^−1^ of N favored the accumulation of stem and root dry biomass, similar to the results of the above study. The aboveground total nitrogen concentration also increased gradually with the increase in N concentration, indicating that 8 mmol·L^−1^ of N could promote the uptake of aboveground total nitrogen concentration and thus, biomass accumulation. The prerequisite for high crop quality and yield is higher biomass accumulation, which is based on nutrient uptake [48]. In this study, the nutrient concentrations of all parts of tomato plants basically showed a trend of increasing and then decreasing with the increase in P concentration. The maximum uptake of nitrogen, phosphorus, and potassium by tomato plants was reached when the amount of P was 1.33 mmol·L^−1^, which followed the same trend as the effect of P on plant leaf biomass. This indicated that 1.33 mmol·L^−1^ of P could promote nutrient uptake and biomass accumulation in tomato plants, similar to the results of Kim [37]. Wang et al. [49] in their study on the effect of potassium fertilizer application on crops showed that an excessive or insufficient supply of K is detrimental to crop nutrient uptake and dry matter mass accumulation. In this study, the K1 level (8 mmol·L^−1^) promoted the accumulation of dry biomass in plant leaves and also had the best effect on total nitrogen and potassium aboveground, total nitrogen and phosphorus belowground, and total potassium in fruits, suggesting that 8 mmol·L^−1^ was a more appropriate K concentration.

Nitrogen, phosphorus, and potassium not only create a favorable growing environment for the formation of vegetable crops but also increase tomato yields. In this study, single fruit weight was maximized at a N concentration of 2 mmol·L^−1^ and suppressed at either 4 or 8 mmol·L^−1^; this may be due to the fact that high nitrogen levels promoted the nutritive growth of tomato plants, which reduced the single fruit weight of tomato fruits [50]. Different ratios of nitrogen, phosphorus, and potassium had different effects on the single fruit weight and yield of tomato in this study. The highest yield, 50,661.46 Kg·hm^−2^, was obtained when the nutrient solution N, P, and K ratio was 8:2:12, which was inconsistent with the studies of Zhang [51] and Zhang [52], which might be mainly closely related to the stubble arrangement, facility environment, and different varieties of experimental tomatoes. Numerous studies have shown that optimizing the ratio of nitrogen, phosphorus, and potassium (NPK) can significantly increase tomato yield, but the optimal ratio varies due to differences in varieties, facility environments, substrate types, and other factors. For example, some studies have found that the N2P2K1 combination (approximately 197:89:230 kg/hm^2^) results in the best yield and quality [53], while in organic substrates the highest tomato yield is achieved when the N:P:K ratio is 350:100:400 g/m^3^ [54].

### 4.4. Tomato Quality and Differential Metabolites

In this study, both 2 and 4 mmol·L^−1^ of N promoted the quality indexes, and 8 mmol·L^−1^ of N inhibited all quality indexes. Wright et al. [53] showed that soluble sugar and total VC concentration increased and fruit quality improved when the N fertilization supply was reduced from 12 to 6 or 4 mmol·L^−1^. In this study, 0.67 mmol·L^−1^ of P promoted the accumulation of VC and soluble solids, and when P exceeded 0.67 mmol·L^−1^ both VC and soluble solids concentrations were suppressed. Wang et al. [54] showed that soluble solids concentration in crops was positively correlated with phosphorus concentration, which is not consistent with the results of the present study and may be due to the use of different crop species. The best effect of 12 mmol·L^−1^ of K was on soluble sugars, VC, soluble solids, and organic acids, and both high potassium and 1.5-fold high potassium inhibited the quality indexes, similar to the findings of Ma [55] and Almeselmani et al. [12]. In addition, metabolomics analysis revealed that Ferulic acid, Cinnamic acid, Caffeic acid, coumarin, and (-)-Epigallocatechin, which are closely related to quality, were significantly increased in both phenylpropane and polyketone compounds in the T2 treatment compared to the T8 treatment. Therefore, it is possible that the increase in the concentration of these substances led to the higher overall quality of the T2 treatment than that of T8, similar to the findings of Zhao [56] and Liao [57].

### 4.5. Analysis of the Effect of Different Levels of N, P, and K on Soil Indicators

For soil nutrients, pH, and EC, when N was increased from 2 to 8 mmol·L^−1^ the soil total and available N concentration increased and most other nutrients decreased. When K increased from 8 to 16 mmol·L^−1^, soil total nitrogen, total phosphorus, and available phosphorus concentration decreased, but available potassium concentration increased and the above N and K concentrations all resulted in higher soil EC values. The reason for these results may be that over-application of N and K has resulted in the over-nutrition of nitrogen and potassium in the soil, resulting in deficiencies in other nutrients and an increase in EC; it may also be due to the fact that pH reached its maximum at 8 mmol·L^−1^ N and soil alkalinization was relatively the most severe, which resulted in the unavailability or deposition of most of the nutrients, similar to the results of Xiao’s [58] study. Soil enzymes, soil catalase, urease, alkaline phosphatase, and sucrase activities were maximized at a N concentration of 8 mmol·L^−1^, and sucrase activities increased at a K concentration of 16 mmol·L^−1^. Yang et al. [59] showed a significant decrease in soil catalase, urease, and sucrase activities under alkali stress, which is inconsistent with the results of the present study and may be due to the difference in a number of factors such as soil temperature, humidity, and microbial population. Soil nutrient concentration and soil enzyme activity were at their maximum and the pH value was at its minimum when the concentration of P was 0.67 mmol·L^−1^, indicating that 0.67 mmol·L^−1^ of P element was more suitable for this soil environment. A highly significant or significant correlation between some soil nutrients and soil enzymes was obtained from the Pearson correlation score, similar to the results of Liu [60]. Different environmental factors such as temperature, humidity, and soil pH affect the correlation between soil enzymes and soil nutrients.

## 5. Conclusions

In this experiment the effects of different N, P, and K ratios in the nutrient solution under brackish water irrigation on the physiological indexes of tomato plants, yields, and quality of tomato fruits, and on soil indexes were analyzed.

(1) N at 8 mmol·L^−1^ was able to further promote vegetative growth of plant and plant biomass accumulation by promoting the accumulation of aboveground nitrogen content but reduced single fruit weight and tomato quality indicators by promoting the vegetative growth of plants. At the same time, this concentration caused most of the soil nutrient concentration to decrease and soil EC and pH to increase, but it can promote soil enzyme activity. For leaf resistance indexes, this concentration was able to suppress MDA concentration while increasing Pro concentration.
(2) Amounts of 0.67 mmol·L^−1^ of P and 12 mmol·L^−1^ of K were able to promote both the vegetative growth of plants and tomato quality formation; however, they were unfavorable to the accumulation of plant nutrients and biomass. The 1.33 mmol·L^−1^ of P promoted both plant nutrients and biomass, and for the index of resistance this concentration was able to promote POD, Pro, and CAT, although it did not inhibit MDA.
(3) Although 0.67 mmol·L^−1^ of P increased soil EC, it was beneficial to soil nutrients, soil enzyme activities, and soil pH. Although 16 mmol·L^−1^ of K increased soil available potassium concentration it was inhibitory to most other nutrients and to soil peroxidase and urease, and it also led to an increase in soil EC. K at 16 mmol·L^−1^ had an inhibitory effect on the MDA concentration in the leaves, while being able to increase the Pro concentration, and this concentration had little effect on SOD, POD, and CAT.
(4) From the metabolomics analysis it can be seen that the overall quality of the T2 treatment was higher than that of the T8 treatment due to the significant increase in the concentrations of Ferulic acid, Cinnamic acid, Caffeic acid, Coumarin, and (-)-Epigallocatechin, which are closely related to quality. And from PCA the T1 treatment had the best overall evaluation and the T5 treatment had the worst overall evaluation.

## Figures and Tables

**Table 1:** N, P, and K concentrations for each treatment.

Treatments	Nutrient Concentrations (Unit: mmol·L^−1^)		
	N	P	K
T1	2.00	0.67	8.00
T2	2.00	1.33	12.00
T3	2.00	2.00	16.00
T4	4.00	0.67	12.00
T5	4.00	1.33	16.00
T6	4.00	2.00	8.00
T7	8.00	0.67	16.00
T8	8.00	1.33	8.00
T9	8.00	2.00	12.00

**Table 2:** Brackish water irrigation schedule.

Reproductive Period	Date	Number of Irrigations	Time per Irrigation (min)
Seedling stage	22 August 2022–18 September 2022	13	10
Flowering and fruiting period	18 September 2022–2 October 2022	14	7
Fruiting stages	2 October 2022–1 January 2023	24	7

**Table 3:** Physical and chemical properties and nutrient concentration of tested soil.

Sample	Physicochemical Property				Nutrient Concentration					
	pH	EC (mS·cm^−1^)	Bulk Weight (g·cm^−3^)	Total Porosity (%)	Total N (g·kg^−1^)	Available N (mg·kg^−1^)	Total P (g·kg^−1^)	Available P (mg·kg^−1^)	Available K (mg·kg^−1^)	Organic Matter (g·kg^−1^)
Soil	7.84	0.67	1.01	61.89	0.35	15.60	0.88	54.78	163.5	20.74

**Table 4:** Growth indicators and root vigor of tomato under different treatments.

Treatments	Plant Height (cm)	Stem Thickness (mm)	Leaf Area (cm^2^)	Root Vigor (µg·g^−1^·h^−1^)
T1	132.2 ± 0.4 abc	12.05 ± 0.15 b	1278.6 ± 46.28 ab	270.3 ± 1.5 ab
T2	131.43 ± 0.35 bc	11.7 ± 0.15 c	1245.41 ± 50.55 abc	304.09 ± 9.28 a
T3	119.83 ± 0.22 d	11.96 ± 0.14 bc	1206.2 ± 47.34 abcd	197.63 ± 9.85 c
T4	134.63 ± 0.73 a	12.44 ± 0.12 a	1168.46 ± 16.01 bcde	233.07 ± 6.4 bc
T5	130.2 ± 0.66 c	11.91 ± 0.14 bc	1068.98 ± 11.68 de	191.29 ± 7.34 c
T6	132.8 ± 0.89 abc	11.81 ± 0.14 bc	1131.93 ± 23.14 cde	240.22 ± 7.61 abc
T7	131.8 ± 0.31 abc	12.09 ± 0.13 b	1310.58 ± 58.98 a	204.99 ± 9.82 bc
T8	131.97 ± 0.97 abc	11.18 ± 0.13 d	1201.75 ± 61.45 abcd	173.52 ± 12.62 cd
T9	133.7 ± 0.68 ab	11.83 ± 0.09 bc	1054.51 ± 35 e	119.13 ± 5.28 d

The values in the table are “mean ± standard deviation” and means followed by a different letter within column are significantly different at *p* < 0.05.

**Table 5:** Leaf biochemical indicators under different treatments.

Treatments	MDA µmol/g FW	SOD U·mg^−1^·min^−1^	POD U·(gF·min^−1^)^−1^	Pro % (Pro)	CAT U·g^−1^·min^−1^
T1	0.81 ± 0.03 b	0.34 ± 0.02 a	1450 ± 17.35 d	0.025 ± 0.001 ef	1910.78 ± 15.94 ab
T2	0.81 ± 0.01 b	0.30 ± 0.02 ab	1558.33 ± 12.73 c	0.024 ± 0.002 ef	1938.25 ± 24.58 a
T3	0.8 ± 0.04 b	0.29 ± 0.03 ab	1466.67 ± 8.33 d	0.03 ± 0.003 cde	1910.02 ± 28.13 ab
T4	0.94 ± 0.02 a	0.26 ± 0.03 b	958.33 ± 8.33 g	0.023 ± 0.001 f	1880.05 ± 33.49 abc
T5	0.76 ± 0.02 b	0.27 ± 0.01 ab	1447.22 ± 5.56 d	0.061 ± 0.002 a	1851.88 ± 9.96 bcd
T6	0.91 ± 0.01 a	0.29 ± 0.03 ab	1830.56 ± 7.35 b	0.038 ± 0.001 b	1720.3 ± 8.4 e
T7	0.76 ± 0.01 b	0.30 ± 0.00 ab	1175 ± 9.62 f	0.034 ± 0.003 bc	1735.95 ± 21.61 e
T8	0.8 ± 0.02 b	0.26 ± 0.01 ab	2727.78 ± 36.11 a	0.033 ± 0.002 bcd	1805.98 ± 8.41 d
T9	0.78 ± 0.03 b	0.29 ± 0.04 ab	1275 ± 8.33 e	0.027 ± 0.002 def	1829.77 ± 14.05 cd

The values in the table are “mean ± standard deviation” and means followed by a different letter within column are significantly different at *p* < 0.05.

**Table 6:** Yield and plant biomass under different treatments.

Treatments	Single Fruit Weight (g)	Yield Per Plant (kg)	Yield Per Hectare (kg/ha)	Fresh Weight (g/Plant)			Mass Fraction of Dry Matter %			
				Leaf	Stem	Root	Fruit	Leaf	Stem	Root
T1	93.16 ± 3.81 ab	1.58 ± 0.13 a	45,526.56 ± 3648.98 a	514.02 ± 52.07 b	267.6 ± 18.98 ab	22.21 ± 0.32 ab	6.1 ± 0.07 bc	9.87 ± 0.02 cd	9.2 ± 0.45 b	13.45 ± 0.38 a
T2	93.39 ± 3.25 ab	1.51 ± 0.12 a	43,449.52 ± 3379.82 a	621.56 ± 63 ab	279.88 ± 16.12 ab	20.31 ± 1.23 b	6.82 ± 0.23 a	10.02 ± 0.33 bcd	9.66 ± 0.36 ab	15.55 ± 0.96 a
T3	93.7 ± 2.55 ab	1.71 ± 0.06 a	49,334.46 ± 1663.47 a	445.98 ± 41.78 b	209.6 ± 29.26 b	21.44 ± 1.59 ab	5.2 ± 0.17 d	11.68 ± 0.42 a	10.89 ± 0.75 ab	13.79 ± 1.07 a
T4	90.79 ± 8.11 ab	1.46 ± 0.11 a	41,992.71 ± 3096.49 a	441.76 ± 63.67 b	199.0 ± 4.37 b	21.53 ± 2.41 ab	5.9 ± 0.04 bc	10.3 ± 0.62 abcd	9.66 ± 0.3 ab	14.12 ± 0.47 a
T5	85.91 ± 4.44 b	1.57 ± 0.08 a	45,387.13 ± 2268.99 a	584.61 ± 30.7 b	265.28 ± 2.48 ab	28.6 ± 2.28 a	5.77 ± 0.44 bcd	10.74 ± 0.39 abc	10.7 ± 0.36 ab	15.27 ± 0.58 a
T6	101.13 ± 8.72 ab	1.65 ± 0.13 a	47,733.41 ± 3700.26 a	622.82 ± 82.28 ab	281.75 ± 32.5 ab	23.07 ± 0.06 ab	6.23 ± 0.09 ab	10.85 ± 0.2 abc	9.95 ± 0.3 ab	13.5 ± 0.26 a
T7	106.21 ± 4.92 a	1.65 ± 0.11 a	47,699.75 ± 3051.58 a	596.65 ± 39.66 ab	267.38 ± 45.39 ab	28.22 ± 3 a	5.62 ± 0.27 bcd	11.47 ± 0.82 ab	11.08 ± 0.44 ab	15.0 ± 0.39 a
T8	102.61 ± 0.78 ab	1.54 ± 0.13 a	44,329.38 ± 3659.12 a	517.98 ± 29.72 b	219.87 ± 28.02 b	24.09 ± 2.03 ab	6.11 ± 0.17 bc	11.63 ± 0.61 a	11.08 ± 1.04 ab	16.09 ± 1.37 a
T9	105.18 ± 7.81 a	1.76 ± 0.07 a	50,661.46 ± 2151.2 a	858.15 ± 49.74 a	317.79 ± 20.76 a	28.37 ± 2.92 a	5.48 ± 0.13 cd	8.99 ± 0.4 d	11.15 ± 0.58 a	16.17 ± 1.08 a

The values in the table are “mean ± standard deviation” and means followed by a different letter within column are significantly different at *p* < 0.05.

**Table 7:** Plant and fruit nutrient concentration under different treatments.

Part	Treatments	N (g·kg^−1^)	P (g·kg^−1^)	K (g·kg^−1^)
Underground part	T1	20.31 ± 0.06 d	0.26 ± 0.01 ef	21.63 ± 0.17 bc
	T2	19.05 ± 0.30 e	0.28 ± 0 de	22.2 ± 0.3 ab
	T3	18.24 ± 0.31 ef	0.22 ± 0.00 g	22.4 ± 0.34 a
	T4	21.91 ± 0.16 c	0.23 ± 0.01 fg	20.36 ± 0.24 de
	T5	27.35 ± 0.15 a	0.43 ± 0.01 a	21.4 ± 0.24 c
	T6	25.49 ± 0.53 b	0.36 ± 0.01 b	18.64 ± 0.14 f
	T7	18.12 ± 0.23 f	0.22 ± 0.01 g	20.61 ± 0.23 d
	T8	19.01 ± 0.32 ef	0.32 ± 0.00 c	19.87 ± 0.19 e
	T9	18.98 ± 0.19 ef	0.31 ± 0.03 cd	18.71 ± 0.1 f
Aboveground part	T1	26.28 ± 0.56 cd	0.28 ± 0.01 cd	31.69 ± 0.15 a
	T2	23.93 ± 0.97 e	0.37 ± 0.01 b	31.49 ± 0.23 a
	T3	22.4 ± 0.13 f	0.29 ± 0.01 cd	30.44 ± 0.07 b
	T4	25.46 ± 0.07 cde	0.38 ± 0.00 b	25.76 ± 0.32 f
	T5	26.54 ± 0.32 bc	0.29 ± 0.00 c	25.63 ± 0.62 f
	T6	24.94 ± 0.17 de	0.25 ± 0.01 d	26.04 ± 0.14 ef
	T7	25.38 ± 0.24 cde	0.28 ± 0.01 cd	26.81 ± 0.24 e
	T8	28.56 ± 0.68 a	0.26 ± 0.01 cd	27.71 ± 0.17 d
	T9	27.96 ± 0.45 ab	0.43 ± 0.01 a	29.44 ± 0.18 c
Fruit	T1	31.27 ± 0.32 c	0.53 ± 0.02 c	34.91 ± 0.1 c
	T2	29.82 ± 0.04 d	0.53 ± 0.01 c	35.77 ± 0.15 b
	T3	34.48 ± 0.04 a	0.59 ± 0.00 a	36.57 ± 0.13 a
	T4	29.22 ± 0.33 d	0.45 ± 0.01 e	34.88 ± 0.26 c
	T5	32.39 ± 0.34 b	0.55 ± 0.02 bc	35.47 ± 0.31 bc
	T6	25.46 ± 0.19 f	0.48 ± 0.01 de	36.96 ± 0.3 a
	T7	31.08 ± 0.36 c	0.51 ± 0.01 cd	32.77 ± 0.09 e
	T8	25.29 ± 0.10 f	0.52 ± 0.02 c	34.72 ± 0.5 c
	T9	27.19 ± 0.23 e	0.58 ± 0.01 ab	33.84 ± 0.22 d

The values in the table are “mean ± standard deviation” and means followed by a different letter within column are significantly different at *p* < 0.05.

**Table 8:** Quality indexes and comprehensive ranking of tomato under different treatments.

Treatments	Soluble Solid/(%)	Vitamin C/(mg·100g^−1^·FW)	Total Soluble Sugar/(%)	Titratable Acid/(%)	Combined PCA Values	Comprehensive Ranking
T1	6.80 ± 0.1 c	18 ± 0.45 abcd	10.13 ± 0.33 bcd	0.57 ± 0.01 a	0.34	4
T2	7.97 ± 0.03 a	19.39 ± 0.25 a	12.66 ± 0.29 a	0.57 ± 0.02 a	1.92	1
T3	6.50 ± 0.06 de	16.57 ± 0.18 de	9.48 ± 0.54 d	0.55 ± 0.02 ab	−0.46	7
T4	7.43 ± 0.07 b	18.55 ± 0.84 abc	11.1 ± 0.36 b	0.55 ± 0.02 ab	0.9	2
T5	6.60 ± 0.1 cd	16.90 ± 0.35 cde	9.97 ± 0.2 cd	0.50 ± 0.01 bc	−0.6	8
T6	6.40 ± 0.1 de	19.41 ± 0.73 a	12.74 ± 0.24 a	0.50 ± 0.01 cd	0.45	3
T7	6.67 ± 0.07 cd	18.74 ± 0.83 ab	10.82 ± 0.22 bc	0.44 ± 0.01 d	−0.42	6
T8	5.57 ± 0.12 f	15.29 ± 0.22 e	8.30 ± 0.22 e	0.49 ± 0.01 c	−2.03	9
T9	6.27 ± 0.09 e	17.15 ± 0.4 bcd	10.15 ± 0.32 bcd	0.58 ± 0.01 a	−0.09	5

The values in the table are “mean ± standard deviation” and means followed by a different letter within column are significantly different at *p* < 0.05.

**Table 9:** Soil nutrients, and EC under different treatments.

Treatments	Total N (g·kg^−1^)	Total P (g·kg^−1^)	Available N (mg·kg^−1^)	Available P (mg·kg^−1^)	Available K (mg·kg^−1^)	Organic Matter (g·kg^−1^)	EC (ms/cm)	pH
T1	0.42 ± 0 bc	0.9 ± 0.04 b	15.98 ± 0.67 bc	747.42 ± 8.85 cd	269.62 ± 2.38 c	27.65 ± 0.2 c	0.25 ± 0.01 b	7.88 ± 0.01 d
T2	0.36 ± 0.01 d	0.74 ± 0.01 e	15.8 ± 0.37 bc	759.12 ± 10.52 bcd	271.73 ± 10.46 c	23.11 ± 0.34 d	0.24 ± 0.01 b	7.86 ± 0.03 d
T3	0.36 ± 0.01 d	0.85 ± 0 c	14.5 ± 0.65 c	762.81 ± 5.57 bcd	324.6 ± 3.59 a	27.62 ± 0 c	0.26 ± 0 b	7.93 ± 0.01 c
T4	0.41 ± 0.01 c	0.8 ± 0.01 d	18.6 ± 0.19 a	815.9 ± 4.75 a	221.36 ± 5.6 de	32.99 ± 0.2 a	0.3 ± 0.02 a	7.84 ± 0 d
T5	0.34 ± 0 e	0.38 ± 0.01 f	16.92 ± 0.67 ab	593.08 ± 3.03 f	233.63 ± 3.24 d	29.04 ± 0.34 b	0.26 ± 0.01 b	7.86 ± 0.01 d
T6	0.37 ± 0.01 d	0.81 ± 0 cd	16.55 ± 0.81 b	736.7 ± 6.07 d	203.17 ± 3.59 f	29.04 ± 0.34 b	0.26 ± 0 b	7.78 ± 0 e
T7	0.38 ± 0 d	0.79 ± 0.01 d	17.29 ± 0.32 ab	686.81 ± 14.54 e	287.68 ± 0.35 b	27.46 ± 0.2 c	0.32 ± 0.01 a	7.85 ± 0.01 d
T8	0.45 ± 0.01 a	0.97 ± 0.01 a	17.48 ± 0.49 ab	778.36 ± 10.13 b	187.88 ± 3.31 g	32.2 ± 0.39 a	0.21 ± 0.01 c	8.02 ± 0 a
T9	0.44 ± 0 ab	0.79 ± 0.01 d	16.36 ± 0.99 bc	765.3 ± 3.37 bc	209.89 ± 3.84 ef	27.06 ± 0.2 c	0.25 ± 0.01 b	7.98 ± 0.01 b

The values in the table are “mean ± standard deviation” and means followed by a different letter within column are significantly different at *p* < 0.05.

**Table 10:** Soil enzyme activities under different treatments.

Treatments	CAT (mg·g^−1^)	Urease (mg·g^−1^)	Phosphatase (mg·g^−1^)	Sucrase (mg·g^−1^)
T1	0.87 ± 0 cde	6.52 ± 0.15 b	0.95 ± 0.05 e	11.31 ± 0.15 c
T2	0.75 ± 0.06 e	3.88 ± 0.27 e	1.08 ± 0.11 de	9.99 ± 0.09 d
T3	0.81 ± 0.06 de	5.97 ± 0.15 bc	1.14 ± 0.09 cde	10.18 ± 0.04 cd
T4	1.1 ± 0.06 b	5.7 ± 0.36 cd	1.5 ± 0.11 a	18.05 ± 0.8 b
T5	0.98 ± 0.06 bc	4.29 ± 0.03 e	1.4 ± 0.01 abc	10.73 ± 0.1 cd
T6	0.92 ± 0.06 cd	5.6 ± 0.03 cd	1.27 ± 0.07 abcd	7.98 ± 0.38 e
T7	0.98 ± 0.06 bc	5.2 ± 0.21 d	1.44 ± 0.03 ab	19.62 ± 0.41 a
T8	1.45 ± 0.06 a	7.05 ± 0.1 a	1.47 ± 0.1 a	7.66 ± 0.2 e
T9	0.87 ± 0 cde	4.4 ± 0.18 e	1.19 ± 0.1 bcde	7.74 ± 0.68 e

The values in the table are “mean ± standard deviation” and means followed by a different letter within column are significantly different at *p* < 0.05.

**Table 11:** Principal component eigenvalues and contributions.

Principal Component	Initial Eigen Value			Extract Sum of Squares and Load		
	Total	Variance	Accumulation	Total	Variance	Accumulation
1	8.107	26.152	26.152	8.107	26.152	26.152
2	5.496	17.730	43.882	5.496	17.730	43.882
3	5.101	16.453	60.335	5.101	16.453	60.335
4	3.696	11.922	72.257	3.696	11.922	72.257
5	3.502	11.295	83.553	3.502	11.295	83.553
6	2.385	7.694	91.247	2.385	7.694	91.247
7	1.502	4.847	96.093	1.502	4.847	96.093
8	1.211	3.907	100.000	1.211	3.907	100.000

**Table 12:** Principal component values and composite rankings of relevant indicators.

Treatments	F1	F2	F3	F4	F5	F6	F7	F8	F	Sort
T1	3.24	1.32	0.87	0.74	1.43	−0.24	−0.35	−2.34	1.35	1
T2	4.36	−1.15	0.96	−1.32	0.47	1.27	1.88	0.98	1.22	2
T3	2.02	2.70	−2.96	1.94	0.54	0.07	−1.13	1.40	0.82	3
T4	0.02	−1.68	3.39	0.80	−2.08	1.23	−1.81	0.35	0.15	4
T5	−1.83	−3.23	−3.87	−0.95	0.12	1.57	−0.33	−0.78	−1.72	9
T6	−1.12	−2.54	0.92	−0.51	2.51	−2.77	−0.47	0.68	−0.58	8
T7	−1.03	−0.61	−0.61	2.60	−2.75	−1.56	1.63	−0.31	−0.53	7
T8	−5.01	2.45	1.60	0.53	1.77	1.48	0.87	0.12	−0.19	5
T9	−0.65	2.74	−0.30	−3.83	−2.03	−1.05	−0.29	−0.10	−0.52	6

## Data Availability

Data are contained within the article.
